# Immunogenicity and Safety of mRNA Anti-SARS-CoV-2 Vaccines in Patients with Systemic Lupus Erythematosus

**DOI:** 10.3390/vaccines10081221

**Published:** 2022-07-30

**Authors:** Ilaria Mormile, Francesca Della Casa, Angelica Petraroli, Alessandro Furno, Francescopaolo Granata, Giuseppe Portella, Francesca Wanda Rossi, Amato de Paulis

**Affiliations:** 1 Department of Translational Medical Sciences, University of Naples Federico II, 80131 Naples, Italy; ilariamormile@virgilio.it (I.M.); francesca.dellacasa@unina.it (F.D.C.); petrarol@unina.it (A.P.); alessandro.furno@unina.it (A.F.); francescopaolo.granata@unina.it (F.G.); portella@unina.it (G.P.); depaulis@unina.it (A.d.P.); 2 Center for Basic and Clinical Immunology Research (CISI), University of Naples Federico II, 80131 Naples, Italy; 3 WAO Center of Excellence, 80131 Naples, Italy

**Keywords:** anti-SARS-CoV-2 mRNA vaccines, COVID-19, SARS-CoV-2, systemic lupus erythematosus, vaccination

## Abstract

Vaccination is the most effective preventive measure to control the spread of COVID-19 and reduce associated complications. This study aims to evaluate the efficacy and safety of mRNA COVID-19 vaccines in patients with systemic lupus erythematosus (SLE). A total of 41 adult SLE patients receiving two doses of the SARS-CoV-2 mRNA Comirnaty-BioNTech/Pfizer vaccine were enrolled. The quantitative determination of anti-trimeric spike protein-specific IgG antibodies to SARS-CoV-2 was assessed before (T0), 21 days after the administration of the first dose of the vaccine (T1), and between 21 and 28 days after the second dose (T2). They were compared with the same determinations from a cohort of 29 patients with C1-esterase inhibitor deficiency hereditary angioedema (C1-INH-HAE) as controls. All the SLE patients and controls demonstrated a positive serological response after a single dose of the vaccine (T1), which significantly increased after the second dose (T2). No significant difference was found between SLE patients and controls at T1 [t(52.81) = −0.68; *p* = 0.49] and at T2 [t(67.74) = −0.22; *p* = 0.825]. Systemic Lupus Erythematosus Disease Activity Index (SLEDAI) analysis showed that the vaccine did not influence SLE activity or caused disease flare in our cohort. In conclusion, COVID-19 vaccines produced a satisfactory response in SLE patients without variation in the disease activity.

## 1. Introduction

Systemic lupus erythematosus (SLE) is an autoimmune disease with chronic or relapsing and remitting disease course, characterized by the loss of self-tolerance and formation of nuclear autoantigens and immune complexes resulting, in inflammation of multiple organs, including the skin, kidneys, joints, and nervous system [1,2]. SLE patients are particularly susceptible to infections, one of the diseases leading causes of morbidity and mortality, due to the underlying autoimmune disorder and the administration of immunosuppressive treatment [3]. Hematological alterations, especially lymphopenia and neutropenia, frequently occur in patients with SLE and may have clinical consequences [3]. In addition, other impaired immune functions have been reported in patients with SLE [4,5]. A decreased number of T lymphocytes and an impaired T-helper cell activity against viral antigens, toxoids, and alloantigens have been observed in SLE patients and correlated with higher disease activity and a longer exposition to glucocorticoids [4]. Complement dysfunction [6] and macrophage-monocyte system dysfunctions, including phagocytosis and oxidative metabolism, are also common in patients with SLE, thus increasing the risk of infections [7].

The current 2019 Coronavirus Disease Pandemic-19 (COVID-19), caused by the severe acute respiratory syndrome coronavirus 2 (SARS-CoV-2), began in December 2019 in Wuhan, China, and quickly became a global health and economic emergency taking an unprecedented burden on the health system around the world [8]. SARS-CoV-2 infection raised particular concern in SLE patients for the higher risk of contracting the disease and potentially evolving to a worse prognosis. Indeed, the immunological alterations (e.g., defects in T cell-mediated cytotoxicity and DNA methylation) observed in SLE may increase patients’ susceptibility to COVID-19 predisposing them to more severe disease [3,9,10]. Data from the COVID-19 Global Rheumatology Alliance physician-reported registry showed that 56% of 85 patients with SLE required hospitalization during COVID-19 infection [11].

Conversely, the increased type I interferon signature characteristic of SLE could enhance protection in the early phases of SARS-CoV-2 infection [12]. In a survey of 126 Italian SLE patients, the COVID-19 incidence was higher than the general population, but the symptoms remained mild to moderate [13]. Accordingly, in other research studies analyzing SLE patients from other countries, most patients showed low COVID-19 incidence and self-resolving symptoms [14]. Overall, the impact of COVID-19 on SLE remains to be explored, considering modifying factors for SLE-induced organ damage and the type of medication used. The use of glucocorticoids and some immunosuppressive drugs commonly used to treat SLE (e.g., rituximab, azathioprine, cyclophosphamide, and calcineurin inhibitors) has been associated with increased COVID-19-related mortality compared with methotrexate [3,15,16]. On the contrary, hydroxychloroquine, beyond its immunomodulatory properties that have encouraged its application in the treatment of autoimmune diseases, exerts antiparasitic, antibacterial, antifungal, and antiviral effects [17]. Indeed, chloroquine shows direct antiviral effects, inhibiting pH-dependent steps of the replication of several viruses, including members of the flaviviruses, retroviruses, and coronaviruses [17].

Vaccination is the most useful preventive measure to control the spread of COVID-19 and reduce associated complications. Patients at risk of more severe disease evolution, such as those receiving immunosuppressive therapy for chronic autoimmune disease and inborn errors of immunity, should be prioritized for vaccination since they could be at a higher risk of severe manifestations of COVID-19 [18]. For this reason, SLE patients have been identified as a priority category for vaccination [19].

Large-scale studies evaluating the immunogenicity of the vaccine against COVID-19 in immunosuppressed patients with autoimmune diseases are currently lacking. In addition, data assessing the safety of the anti-SARS-CoV-2 vaccine in this type of patient and its potential effect on the underlying disease flares are needed. mRNA vaccines represent a new vaccine technology leading to increasing insecurity, especially in patients with immune system disorders. Severe allergic vaccination reactions are rare and can be caused by the vaccine itself (very rare) or the ingredients (adjuvants, antibiotics, hen’s egg, carriers, or preservatives) of the vaccine solution [18].

We aim to evaluate the real-life anti-SARS-CoV-2 vaccine efficacy and safety, assessing how people with SLE respond to COVID-19 vaccination to provide valuable information about benefits and potential risks in these individuals. In particular, the primary outcome was to assess the persistence of vaccine-induced antibodies after two doses of the COVID-19 vaccine compared to controls. Secondary outcomes included collecting information about vaccine safety in people with SLE and exploring, in particular, the possibility of reactivating the underlying disease.

## 2. Materials and Methods

### 2.1. Patients

A total of 41 adult patients diagnosed with SLE at the Division of Allergy and Clinical Immunology of the University of Naples Federico II, Naples, Italy, were enrolled in this prospective observational study. All patients met definitions for at least one of the following: (i) the American College of Rheumatology (ACR) revised classification criteria [20]; (ii) the Systemic Lupus International Collaborating Clinics (SLICC) classification criteria [21]; or (iii) the European League Against Rheumatism (EULAR)/ACR classification criteria [22]. They were aged >18 years, with available data on sex, date of birth, age of onset, SLE diagnosis age, and signature of the written informed consent. Patients with a history of allergic reaction to any component of the vaccine or with a history of severe allergic reaction to any allergens were excluded. At enrollment, participants were asked if they had been diagnosed with COVID-19 in the past and about symptom severity.

Patients enrolled were in treatment with a selection of immunosuppressive therapies. Based on the current treatment, patients were divided into four groups: group 1: no therapy; group 2: low-level immunosuppression (i.e., patients treated with hydroxychloroquine in monotherapy); group 3: medium-level immunosuppression (i.e., azathioprine, methotrexate, prednisone < 20 mg/die, ciclosporin, belimumab given in monotherapy); and group 4: high-level immunosuppression (i.e., prednisone > 20 mg/die, mycophenolate mofetil, cyclophosphamide, combination therapy) [23].

Subjects with C1-esterase inhibitor deficiency hereditary angioedema (C1-INH-HAE) without previous COVID-19 infection or any other significant acute/chronic illness (including autoimmune disease) participated in another study investigating the safety and tolerability of the COVID-19 vaccines in people with hereditary angioedema and were selected as controls. Controls were age and gender-matched with the patients with SLE.

All participants enrolled received two doses of the SARS-CoV-2 mRNA Comirnaty-BioNTech/Pfizer vaccine.

Follow-up study visits were scheduled 7 days before (T0) and 21 days after the administration of the first dose of the vaccine (T1) and between 21 and 28 days after the second vaccine dose (T2).

SLE disease activity was measured using the Safety of Estrogens in Lupus Erythematosus National Assessment (SELENA) version of the Systemic Lupus Erythematosus Disease Activity Index 2000 (SLEDAI-2k) [24,25,26] before the administration of the first dose (T0) and 21 days after the administration of the second dose of the vaccine (T2). At baseline and T2, all SLE patients also had complete blood picture, liver, and renal function tests, C3, C4, anti-nuclear antibodies (ANA), erythrocyte sedimentation rate (ESR), C-reactive protein concentration (CRP), anti-double strain DNA (anti-dsDNA), and urine (routine microscopy and 24-h urine protein test) checked.

All the visits were conducted in person at the Division of Allergy and Clinical Immunology of the University of Naples Federico II. During these multiple follow-up time points, participants were asked about any vaccine-related adverse events to better understand the safety and tolerability of the vaccines in people with SLE. All procedures performed in this study were in accordance with the ethical standards of the study center and with the 1964 Helsinki Declaration and its later amendments or comparable ethical standards. The study protocol was approved by the Ethics Committee of the University Hospital Federico II of Naples, Italy. All the subjects enrolled gave informed consent to participate in the study.

### 2.2. Blood Samples Collection

The patients provided a blood sample to investigators seven days before receiving the first dose of the vaccine (T0). Additional blood samples were collected 21 days after the first dose (T1) and between 21 and 28 days after the second dose of the vaccine (T2).

Blood sampled before and shortly after vaccination was used to study the short-term immunological effects of immunization.

The quantitative determination of anti-trimeric spike protein-specific IgG antibodies to SARS-CoV-2 was quantified by chemiluminescence immunoassay (CLIA) according to the manufacturer protocol (kit LIAISON Ctrl SARS-CoV-2 TrimericS IgG, DiaSorin S.p.A., Saluggia (VC), Italy). This assay provides an indication of the presence of neutralizing IgG antibodies against SARS-CoV-2. The LIAISON^®^ SARS-CoV-2 TrimericS IgG assay measures between 4.81 and 2080 BAU/mL. Test results are reported as positive (≥33.8 BAU/mL) or negative (<33.8 BAU/mL), along with a numeric value for quantitative measurement.

### 2.3. Data Analysis

Data were summarized by descriptive analysis. Means and SD were calculated for continuous variables, while absolute values and frequency (percentage) were calculated for categorical variables. Analysis of anti-SARS-CoV-2-IgG levels was performed with repeated-measures 1-way ANOVA with T0, T1, and T2 visits as a within-subject factor.

Post-hoc comparisons were performed with dependent t-tests corrected with the Sidak procedure. Analysis of dependent variables “anti-SARS-CoV-2-IgG levels” at T1 and T2 was performed with an unpaired t-test comparing the two groups (“SLE patients” vs. “C1-INH-HAE controls”). Analysis of dependent variables “anti-SARS-CoV-2-IgG levels” at T0, T1, and T2 was performed with an unpaired t-test comparing two groups of patients according to the levels of immunosuppression (“no therapy to medium-level immunosuppression-group 1 to 3” vs. “high-level immunosuppression-group 4”). Analysis of dependent variable SLEDAI was performed with Wilcoxon test comparing the two visits (T0 vs. T2). To test the predictive value of SLEDAI on anti-SARS-CoV-2-IgG levels at T2 we used linear regression analysis. A significance level of *p* < 0.05 was assumed for all statistical evaluations. All analyses were performed with IBM SPSS Statistics for Mac, Version 28.0.1.0 (IBM Corp., Armonk, NY, USA).

## 3. Results

### 3.1. Demographics

A total of 41 adult patients [5 males (12.19%) and 36 females (87%)] diagnosed with SLE and 29 controls with C1-INH-HAE [9 males (31.03%) and 20 females (68.96%)] were enrolled in this study. The cohort was White-Caucasian. The average age at enrollment was 45 ± 12 years (19–69). The mean age-of-onset of SLE was 26 ± 11 years. Clinical features in our cohort of patients with SLE are summarized in Table 1.

### 3.2. Clinical Evaluation

Six out of forty-one (14.6%) patients had recovered from mild-to-moderate COVID-19 during the previous months. All the infections have been treated at home with oral glucocorticoids (Prednisone 25 mg/day for two weeks) with subsequent prednisone tapering, azithromycin 500 mg/day, and low molecular weight heparin (LMWH) 4000 UI/day, without invasive or non-invasive ventilation.

We evaluated the influence of vaccine administration on disease activity by assessing the Safety of Estrogens in Lupus Erythematosus National Assessment (SELENA) version of the Systemic Lupus Erythematosus Disease Activity Index 2000 (SLEDAI-2k) at T0 and T2. SLEDAI at T0 was available for 32 patients, while SLEDAI at T2 was available for 26 patients. Mean SLEDAI was 6 ± 6.67 at T0 and 5 ± 6.93 at T2. 14 out of 32 (43.75%) patients had the active disease (score ≥ 6) on SLEDAI at T0, while 8 out of 26 (30.76%) patients had the active disease (score ≥ 6) on SLEDAI at T2 (Table 1). We then evaluated the SLEDAI variation at T2 compared to the baseline for each patient to assess the occurrence of disease flare after the anti-SARS-CoV-2 vaccine. SLEDAI was available at both T0 and T2 for 25 out of 41 (60.97%) patients, of whom 6 (24%) patients presented a SLEDAI increase, 10 (40%) patients a SLEDAI reduction, and 9 (36%) patients did not present any variation between the two SLEDAI assessment. SLEDAI analysis performed with the Wilcoxon test did not reveal a significant difference between the T0 and T2 (*p* = 0.640), showing that the anti-SARS-CoV-2 vaccine did not influence SLE activity or caused disease flare in our cohort. In addition, there was no significant change in ANA, ESR, CRP, anti-dsDNA, and proteinuria after vaccination. The main therapeutic strategies used in our SLE patient cohort are displayed in Table 2.

All patients reported mild and transient adverse reactions after the vaccine administration (i.e., local reaction, headache, muscle pain, joint pain, and tiredness). No patients experienced severe adverse events following the administration of the first or the second dose of the vaccine.

### 3.3. Anti-SARS-CoV-2 Vaccine Immunogenicity

**Figure 1:**
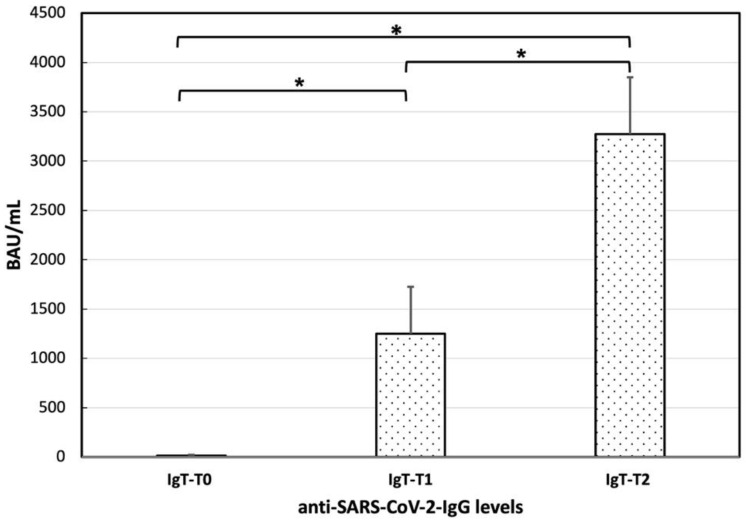
Anti-SARS-CoV-2-IgG levels at T0, T1, and T2 in our systemic erythematous lupus patients (*n* = 41). ANOVA conducted on anti-SARS-CoV-2-IgG levels revealed a significant difference (*) among the determinations at T0, T1, and T2 [F(2,80) = 17.65; *p* < 0.001]. Post-hoc comparisons revealed significantly higher levels of anti-SARS-CoV-2-IgG levels at T1 (post-hoc *p* < 0.05) and T2 (post-hoc *p* < 0.001) compared to T0, and at T2 and compared to T1 (post-hoc *p* < 0.05).

In patients with SLE, mean anti-SARS-CoV-2-IgG levels at T0 were 14.59 ± 60.77. A total of 29 out of 41 patients (70.73%) showed the absence of anti-SARS-CoV-2-IgG at T0. A total of 3 out of 41 (7.31%) patients showed positive anti-SARS-CoV-2-IgG at T0, of whom two patients had recovered from COVID-19 the month earlier, and one did not acknowledge a previous SARS-CoV-2 infection. A total of 9 out of 41 (21.95%) patients presented a very low level of IgG antibodies to the pathogen (range 4.84–17 BAU/mL), which was still below the cut-off positivity level of 33.8 BAU/mL. Among them, two patients had a previous SARS-CoV-2 infection documented by polymerase chain reaction (PCR) test, and seven did not acknowledge a previous SARS-CoV-2 infection. In our cohort, a total of six patients had a prior PCR confirmed diagnosis of COVID-19, of whom two showed the absence of anti-SARS-CoV-2-IgG at T0, two had a very low level of IgG antibodies, and two positive anti-SARS-CoV-2-IgG at T0. Mean anti-SARS-CoV-2-IgG levels were 1248.95 ± 3046.86 at T1 and 3273.40 ± 3679.57 at T2 (Figure 1).

**Figure 2:**
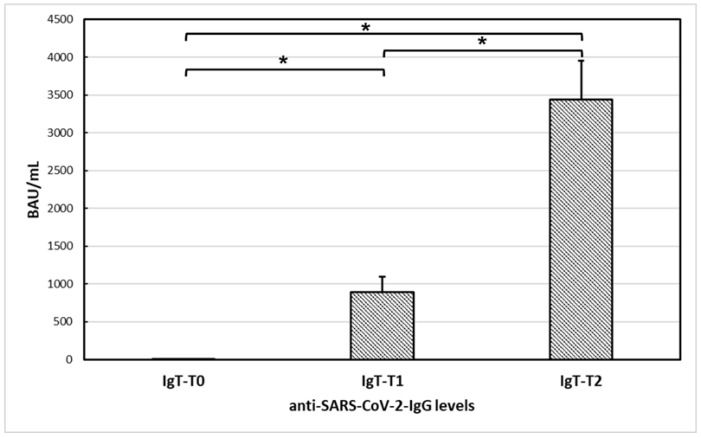
Anti-SARS-CoV-2-IgG levels at T0, T1, and T2 in controls with C1-esterase inhibitor deficiency hereditary angioedema (*n* = 29). ANOVA conducted on anti-SARS-CoV-2-IgG levels revealed a significant difference (*) among the determinations at T0, T1, and T2 [F(1,27) = 39.21; *p* < 0.001]. Post-hoc comparisons revealed significantly higher levels of anti-SARS-CoV-2-IgG levels at T1 (post-hoc *p* < 0.05) and T2 (post-hoc *p* < 0.001) compared to T0, and at T2 and compared to T1 (post-hoc *p* < 0.05).

All the controls with C1-INH-HAE demonstrated a positive serological response after a single dose of the vaccine (T1), which significantly increased after the second dose (T2). Specifically, mean anti-SARS-CoV-2-IgG levels were 2.25 ± 4.26 at T0, 895.76 ± 1067.43 at T1, and 3443.97 ± 2753.72 at T2. Furthermore, ANOVA conducted on anti-SARS-CoV-2-IgG levels of controls with C1-INH-HAE revealed a significant difference among the determinations at T0, T1, and T2 [F(1,27) = 39.21; *p* < 0.001]. Post-hoc comparisons revealed significantly higher levels of anti-SARS-CoV-2-IgG levels at T1 (post-hoc *p* < 0.05) and T2 (post-hoc *p* < 0.001) compared to T0, and at T2 and compared to T1 (post-hoc *p* < 0.05) (Figure 2).

ANOVA conducted on anti-SARS-CoV-2-IgG levels of SLE patients revealed a significant difference among the determinations at T0, T1, and T2 [F(2,80) = 17.65; *p* < 0.001]. Post-hoc comparisons revealed significantly higher levels of anti-SARS-CoV-2-IgG levels at T1 (post-hoc *p* < 0.05) and T2 (post-hoc *p* < 0.001) compared to T0, and at T2 and compared to T1 (post-hoc *p* < 0.05), demonstrating that anti-SARS-CoV-2 vaccine was effective in patients with SLE (Figure 1). In detail, 26 out of 41 (63.41%) patients demonstrated a positive serological response after a single dose of the vaccine (T1), and the vast majority of SLE patients (37 out of 41 patients; 90.24%) showed a positive serological response at T2.

**Figure 3:**
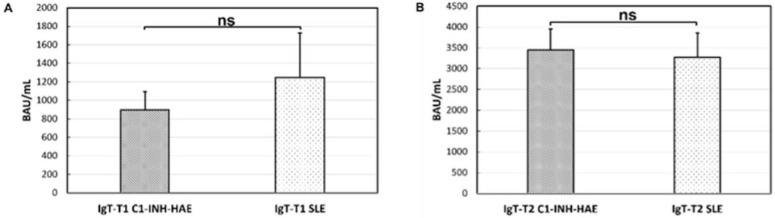
Comparison between anti-SARS-CoV-2-IgG levels at T1 and T2 between systemic erythematous lupus patients (SLE) patients and C1-esterase inhibitor deficiency hereditary angioedema (C1-INH-HAE) controls. No significant difference (ns) was found at T1 [t(52.81) = −0.68; *p* = 0.49] (**A**) and at T2 [t(67.74) = −0.22; *p* = 0.825] (**B**) between the two groups of patients.

Analysis of anti-SARS-CoV-2-IgG levels did not reveal a significant difference between SLE patients and C1-INH-HAE controls at T1 [t(52.81) = −0.68; *p* = 0.49] (Figure 3A) and at T2 [t(67.74) = −0.22; *p* = 0.825] (Figure 3B).

The antibody level was also correlated with SLEDAI. Regression analysis showed that SLEDAI was not a significant predictor of anti-SARS-CoV-2-IgG levels at T2 (R = 0.17; *p* = 0.40).

**Figure 4:**
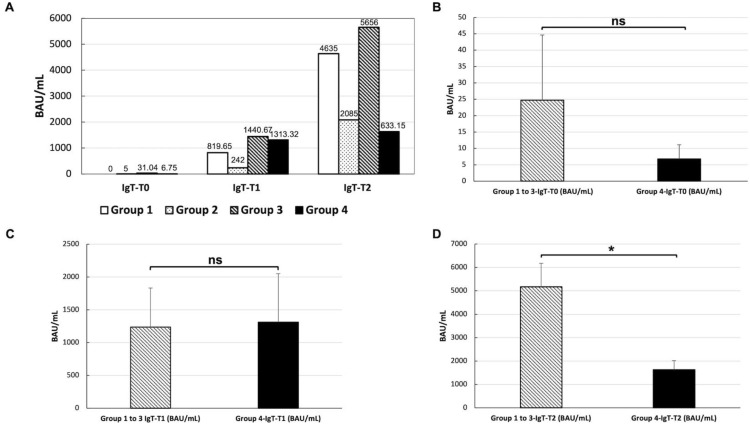
Anti-SARS-CoV-2-IgG levels at T0, T1, and T2 in patients treated with different immunosuppression levels (**A**). Group 1: no therapy; group 2: low-level immunosuppression (i.e., hydroxychloroquine alone); group 3: medium-level immunosuppression (i.e., azathioprine, methotrexate, prednisone < 20 mg/die, ciclosporin, belimumab given in monotherapy); and group 4: high-level immunosuppression (i.e., prednisone > 20 mg/die, mycophenolate mofetil, cyclophosphamide, combination therapy). Comparison between anti-SARS-CoV-2-IgG levels between anti-SARS-CoV-2-IgG levels between patients with no treatment to medium treatment (i.e., group 1 to 3) and with high treatment (i.e., group 4). No significant difference (ns) was found at T0 [t(19) = 0.84; *p* = 0.41] (**B**) nor at T1 [t(36) = −0.22; *p* = 0.82]] (**C**) between the two groups of patients. A significant difference between the two groups (*) was found at T2 [t(22) = 3.41; *p* < 0.002], showing higher anti-SARS-CoV-2-IgG levels for the patients with no treatment to medium treatment (i.e., group 1 to 3) compared with patients with high treatment (**D**).

According to the treatment administrated, patients were stratified into four groups: 2 (4.8%) patients were included in group 1 (no therapy); 2 (4.8%) patients in group 2 (low-level immunosuppression), 14 (34.14%) patients in group 3 (medium-level immunosuppression), and 23 (56.09%) patients in group 4 (high-level immunosuppression). Figure 4A displays the anti-SARS-CoV-2-IgG levels at T0, T1, and T2 in patients belonging to the four groups of immunosuppression, showing that all patients achieved a successful immunization at T2. In order to evaluate the influence of single medications on the immunization of SLE patients, we compared the anti-SARS-CoV-2-IgG levels at T0, T1, and T2 between the combined patients in groups 1, 2, and 3 (i.e., no treatment to medium treatment) and patients in group 4 (i.e., high treatment). No significant difference was found at the baseline [t(19) = 0.84; *p* = 0.41] (Figure 4B) nor at T1 [t(36) = −0.22; *p* = 0.82] (Figure 4C) between the two groups of patients. On the contrary, a significant difference between the two groups was found at T2 [t(22) = 3.41; *p* < 0.002], showing higher anti-SARS-CoV-2-IgG levels for the patients with no treatment to medium treatment compared with patients with high treatment (Figure 4D).

Among the four patients (9.75%) showing absent anti-SARS-CoV-2-IgG at T2, one at the time of enrollment was under treatment with prednisone 10 mg/day and hydroxychloroquine 400 mg/day (group 3), one with mycophenolate mofetil 2 g/day (group 4), and two with prednisone 10–12.5 mg/day, hydroxychloroquine 400 mg/day, and mycophenolate mofetil 2–3 g/day (group 4).

## 4. Discussion

The COVID-19 infection has been troubling the world since early 2020, and vaccination has been considered the only potentially resolving strategy to manage it. From the beginning of the COVID-19 pandemic, growing attention has been focused on protecting some categories at higher risk of infection, such as patients with autoimmune diseases, especially if treated with immunosuppressant drugs. For this reason, SLE patients have been identified as a priority category for vaccination [19]. However, before the COVID-19 pandemic, studies evaluating vaccine immunogenicity in homogenous cohorts of patients with autoimmune diseases were scarce.

In this study, we found that the anti-SARS-CoV-2 vaccine was effective in inducing antibody response in patients with SLE (Figure 1), with no significant difference in the anti-SARS-CoV-2-IgG levels after the first and the second dose between SLE patients and controls (Figure 3). However, results from other SLE patient cohorts have shown opposite findings. A recent study by So et al. [16] analyzed 65 SLE patients receiving two doses of COVID-19 vaccines [i.e., the inactivated vaccine CoronaVac^®^ (Sinovac) and the mRNA-based vaccine Comirnaty^®^ (BioNTech/Fosun)] demonstrated a satisfactory but impaired humoral response in SLE patients compared to controls which were dependent on the immunosuppressive medications use (the use of mycophenolate mofetil and the dosage of systemic corticosteroids were independent predictors of neutralizing antibody levels at day 28 after vaccination) and type of vaccines received. Another study by Izmirly and colleagues [27], analyzing the immunization of SLE patients with three different vaccines (two mRNA and one adenovirus vaccine) compared to healthy controls, described an impaired response in SLE patients. Finally, some authors compared the COVID-19 vaccine immunogenicity between patients with different rheumatic diseases without a healthy control group [28,29,30]. For instance, Simon et al. [29] reported a global 77% response to the mRNA vaccine without a distinction between 64 SLE patients and 73 rheumatoid arthritis patients identifying rituximab as the only drug associated with a pronounced reduction in immunogenicity. The difference in vaccine response in SLE patients may be due also to the type of vaccine used. Indeed, the immunogenicity of the inactivated vaccine has been shown to be substantially lower compared with other vaccine types [31] and more attenuated in patients with autoimmune rheumatic diseases [32]. For these reasons, cumulative evidence recommends a third dose of SARS-CoV-2 vaccination in these patients [33]. Data from SLE patients receiving a heterogeneous booster dose (i.e., mRNA or viral vector vaccine following two doses of inactivated vaccine) showed that humoral and cellular immune response was substantially enhanced after the administration of the booster dose, while all patients had low-positive anti-spike antibodies prior to its administration [33].

To date, little is known about the influence of single medications on SLE patients’ immunization due to the small sample size of the currently published studies, which precluded an accurate analysis of the effects of the individual drugs. Based on a recently published study by Yuki et al. [34], enrolling 232 SARS-CoV-2–naive SLE patients in different immunosuppressive therapies reported that prednisone and mycophenolate mofetil had a significant deleterious effect on vaccine response. On the contrary, hydroxychloroquine may improve anti-SARS-CoV-2 S1/S2 IgG seroconversion, supporting the need to explore the role of temporary mycophenolate mofetil withdrawal or a vaccine-booster dose. In another broad prospective study by Moyon et al. [35], mycophenolate mofetil, methotrexate, and a reduction in naïve B cell pool were independently associated with impaired mRNA vaccine antibody response. These results when taken together aligned with the significatively lower anti-SARS-CoV-2-IgG levels we found in our SLE cohort, among patients with high immunosuppression levels after the second dose of the vaccine (Figure 4D). Indeed, despite all our SLE patients achieving a successful immunization at T2 (Figure 3), some drugs such as mycophenolate mofetil, prednisone > 20 mg/die, cyclophosphamide, and the combination therapy seemed to influence the vaccine immunogenicity more than other treatment. The straightness of these findings is limited by our small sample size and the heterogeneity of the patients combined from groups 1 to 3. However, these observations could be a starting point for multi-center studies, which could assess the influence of single medications on the immunization of SLE patients on a larger scale.

Due to the lack of broad clinical trials evaluating the COVID-19 vaccination safety profile in patients with SLE, there were concerns about the risks of inducing disease flares in patients with underlying autoimmunity. Indeed, immune hyperactivation has been associated with several vaccines [36]. Moreover, new-onset SLE post-COVID-19 immunization is a rare but possible adverse event [37]. The SLEDAI assessment in our cohort showed that the anti-SARS-CoV-2 vaccine did not influence SLE activity or caused disease flares in the short term. Indeed, after the vaccine administration, most of our cohort (19 out of 25 patients, 76%) presented with a decreased or unvaried SLEDAI value. These results aligned with the published literature on the COVID-19 vaccine in SLE patients reporting no evidence of worsening disease after vaccination in the short term [16,27,35]. A broad web-based survey enrolling patients with systemic autoimmune or inflammatory rheumatic diseases, including SLE, reported a medically confirmed flare after vaccination in only 3–4,6% of patients [38].

In conclusion, our results showed that COVID-19 vaccines produced a satisfactory response in SLE patients without variation in the disease activity.

## Figures and Tables

**Table 1:** Clinical features in our cohort of patients with systemic lupus erythematosus (*n* = 41).

		Number	Percentage
**Sex**	M	5	12.19%
	F	36	87.8%
**COVID-19 disease**	Positive	32	78%
	Negative	6	14.63%
	Unknown	3	7.31%
**Antiphospholipids**	Positive	13	31.7%
	Negative	18	43.9%
	Unknown	10	24.39%
**Immunosuppression level**	No therapy	2	4.87%
	Mild	2	4.87%
	Moderate	15	36.58%
	High	22	53.66%
**Disease activity T0 (SLEDAI)**	Inactive disease (0)	12	29.27%
	Mildly active disease (1–5)	7	17.07%
	Moderate actively (6–10)	6	14.63%
	Active disease (11–19)	7	17.07%
	Very active disease (≥20)	1	2.43%
	Not available	8	19.51%
**Disease activity T2 (SLEDAI)**	Inactive disease (0)	10	24.39%
	Mildly active disease (1–5)	9	21.95%
	Moderate actively (6–10)	5	12.19%
	Active disease (11–19)	3	7.32%
	Very active disease (≥20)	1	2.43%
	Unknown	13	31.7%

**Table 2:** Immunosuppressant therapies used in our cohort of patients with systemic lupus erythematosus.

Treatment	Number of Patients (%) Number of Studied Patients = 41
Patient without therapy	2 (4.87%)
HCQ	2 (4.87%)
PDN (<20 mg/day)	4 (9.75%)
MMF	1 (2.44%)
BLM	1 (2.44%)
HCQ + PDN (<20 mg/day)	8 (19.5%)
HCQ + AZA	1 (2.44%)
HCQ + MTX	1 (2.44%)
HCQ + MMF	2 (4.87%)
HCQ + BLM	2 (4.87%)
PDN (<20 mg/day) + MMF	2 (4.87%)
PDN (<20 mg/day) + CYA	1 (2.44%)
PDN (>20 mg/day) + MMF	1 (2.44%)
HCQ + PDN (<20 mg/day) + BLM	5 (12.19%)
HCQ + PDN (<20 mg/day) + MMF	5 (12.19%)
HCQ + PDN (<20 mg/day) + SCIG	1 (2.44%)
HCQ + PDN (>20 mg/day) + BLM	1 (2.44%)
MMF + BLM	1 (2.44%)

HCQ: hydroxychloroquine; PDN: prednisone; MMF: mycophenolate mofetil; BLM: belimumab; AZA: azathioprine; MTX: methotrexate; CYA: cyclosporine A; SCIG: subcutaneous immunoglobulin.

## Data Availability

The data presented in this study are available on request from the corresponding author.
